# Pathological diagnosis of thyroid nodules directly from ultrasonography by a weakly supervised deep learning framework

**DOI:** 10.3389/fendo.2026.1834977

**Published:** 2026-06-01

**Authors:** Xiao-Wen Hou, Mei-Ling He, Meng-Yue Ye, Jian-Xin Ji, Liu-Ying Wang, Wei Zhang, Yue Zhao, Zhen-Zhao Sun, Wei Zhang, Hui-Ru Jiang, Ping Li, Ji-Hong Wang, Fan-Chao Shi, Shu-Xin Sun, Lei Cao

**Affiliations:** 1 Ningbo Hangzhou Bay Hospital, Ningbo, China; 2 Renji Hospital, School of Medicine, Shanghai Jiao Tong University, Shanghai, China; 3 Department of Biostatistics, School of Public Health, Harbin Medical University, Harbin, China; 4 Department of Ultrasonography, The Second Affiliated Hospital of Jiaxing University, Jiaxing, China; 5 Department of Neurology, The Second Affiliated Hospital of Jiaxing University, Jiaxing, China

**Keywords:** attention mechanism, deep learning, pathology, thyroid nodule, ultrasonography

## Abstract

**Background:**

The management of ultrasonography (US) of thyroid nodules is often time consuming and may be inconsistent between observers. The use of deep learning (DL) can increase the speed of examinations and improve reporting consistency. To develop a novel dual attention-guided DL framework to inference histological status directly from thyroid US without expensive image-level annotations.

**Methods:**

In this retrospective analysis, patients first underwent US followed by histopathological examination by fine needle aspiration or surgical histological analysis. Two datasets from the Second Affiliated Hospital of Jiaxing University, Batch 1 and Batch 2, were used in our study for training, testing and validation of the algorithm, respectively. The proposed DL framework is named ThyUS2Path and consists of two attention modules which focus on discriminative nodule patterns in different dimensions. We compare ThyUS2Path with two commonly used state-of-the-art MIL-based methods.

**Results:**

We used 6014 images from 603 patients to build the DL framework and evaluated it on 1978 external images, five-fold cross validation AUCs of 0.754 ± 0.035 and 0.735 ± 0.029, respectively. ThyUS2Path outperformed two commonly state-of-the-art MIL-based methods significantly, namely Maxpool and Meanpool. The algorithms also obtained good prediction performance in the external test set (AUROCs of 0.70~0.80 and AUPRCs of 0.78~0.83).

**Conclusion:**

Our approach gives a feasible way to link the US phenotype with histological reports and has the potential to augment clinicians’ capabilities in thyroid cancer diagnosis in a non-invasive manner.

## Introduction

Diagnostic ultrasonography is the preferred tool for evaluating thyroid nodules using the imaging technique of ultrasound (1–4). Compared to other medical imaging techniques, ultrasound is cost effective, non-invasive, non-radioactive and allows the physician to select the area of interest during image acquisition (5). Microcalcifications, distorted contours or nodules with irregular margins are important ultrasound features of malignant thyroid nodules. The widespread use of imaging methods has contributed to a 2.4-fold increase in the incidence of thyroid cancer over the last 30 years, the fastest increase of any cancer type (6, 7), with a correspondingly unchanged or declining mortality rate for thyroid cancer (8). This has given rise to the need for accurate diagnosis of thyroid cancer. In actual studies, there are overlapping ultrasound image features of benign and malignant thyroid nodules with blurred nodule appearance and irregular shape (9, 10), making manual feature extraction and annotation difficult for experience-based clinicians. In addition, the clinical experience of the operator, and different diagnostic criteria can interfere with the physician’s assessment of thyroid nodules (11, 12). The sensitivity of using US to diagnose thyroid cancer ranges from 27% to 63% only (13, 14). In clinical practice, ultrasound TIRADS stage >3 is recommended to perform fine needle aspiration biopsy (FNAB) or surgical resection (15). However, FNAB is ineffective and suffers from ultrasound localization of nodules, failing to provide a definitive diagnosis in at least 20% of patients or requiring repeated FNABs that still do not yield definitive results (16–19). Therefore, considering the exponentially increasing patient demand and reducing the burden on healthcare services, computer-aided diagnosis was introduced to improve the accuracy of the diagnostic process/hopefully automating image analysis for a robust diagnosis.

Computer-aided diagnosis systems generally consist of three parts: nodule detection, imaging feature extraction, and classifier construction (20). Classical machine learning (ML) methods consist of manually defined features by radiologists and conventional classifiers such as SVM. The process tends to be influenced by operator experience and is also an expensive and/or time-consuming process resulting in models with low discriminatory power (21, 22). Deep learning (DL) is a subset of ML and artificial intelligence and can automatically extract features from images with complex hierarchical structures (23). The introduction of DL such as convolutional neural networks in thyroid imaging has achieved better diagnostic results than experienced radiologists (24–26). However, in practice, thyroid ultrasonography acquires the region of interest by reading dynamic images, the number of which is usually large and uneven. Using a single ultrasound image as a sample to diagnose thyroid nodules will lose the overall information. Besides, different nodules of the same patient may have different labels, and the annotation of all images is a tedious and time-consuming task. Furthermore, the previous DL-based methods tend to be trained using TRADS reports as image labels. After that, histological examinations must be conducted to obtain the final histological diagnosis.

Therefore, we would like to develop a clinically useful diagnostic model that is able to make histological diagnosis of thyroid nodules directly from US. The model, named ThyUS2Path, is robust to different ultrasound images of the same patient without detailed image-level annotations. In this case, multi-instance learning (MIL) is an ideal tool to build a robust classifier on multiple 2D ultrasound images of the same case. MIL is a bag-based model to be trained by considering images originating from each patient as a bag with label. Each image of the bag can be considered as an instance without detailed annotation (27–29). In particular, a dual-attention module is designed and integrated into the neural network to estimate the instance-level importance scores and fuse them to produce bag-level classification result. We trained ThyUS2Path on 6014 thyroid nodule images and validated it on an independent dataset of 1978 images to evaluate its generalizability.

## Materials and methods

### Study design and datasets

Two datasets from the Second Affiliated Hospital of Jiaxing University were used in our study (1): batch1: 6014 thyroid images from 603 patients; (2) batch2: 1978 thyroid images from 108 patients. The histological diagnosis of all these images were available. The datasets were prepared according to the following inclusion criteria: (1) hemi- or total thyroidectomy, (2) maximum nodule diameter 2.5 cm, (3) examination by conventional US and real‐time elastography (RTE) within 1 month before surgery, and (4) no previous thyroid surgery or percutaneous thermotherapy.

Labels were assigned at the patient level in the MIL framework. For patients with multiple nodules, the patient-level label was determined by the most severe histopathological diagnosis (i.e., malignant if any nodule was confirmed malignant). All ultrasound images from the same patient were linked to that patient-level label. We split 603 patients from batch1 dataset into training-validation (90% of patients) and testing sets (10% of patients). Then the training-validation set was split in a five-fold cross validation way. A total of 4389 images and 1077 images were used for training and validation in each fold, respectively. The validation set in each fold was not overlapped. Finally, we obtained a total of 548 images for internal testing, while the other dataset of 1978 images were all for external independent validation.

### The proposed ThyUS2Path architecture

**Figure 1:**
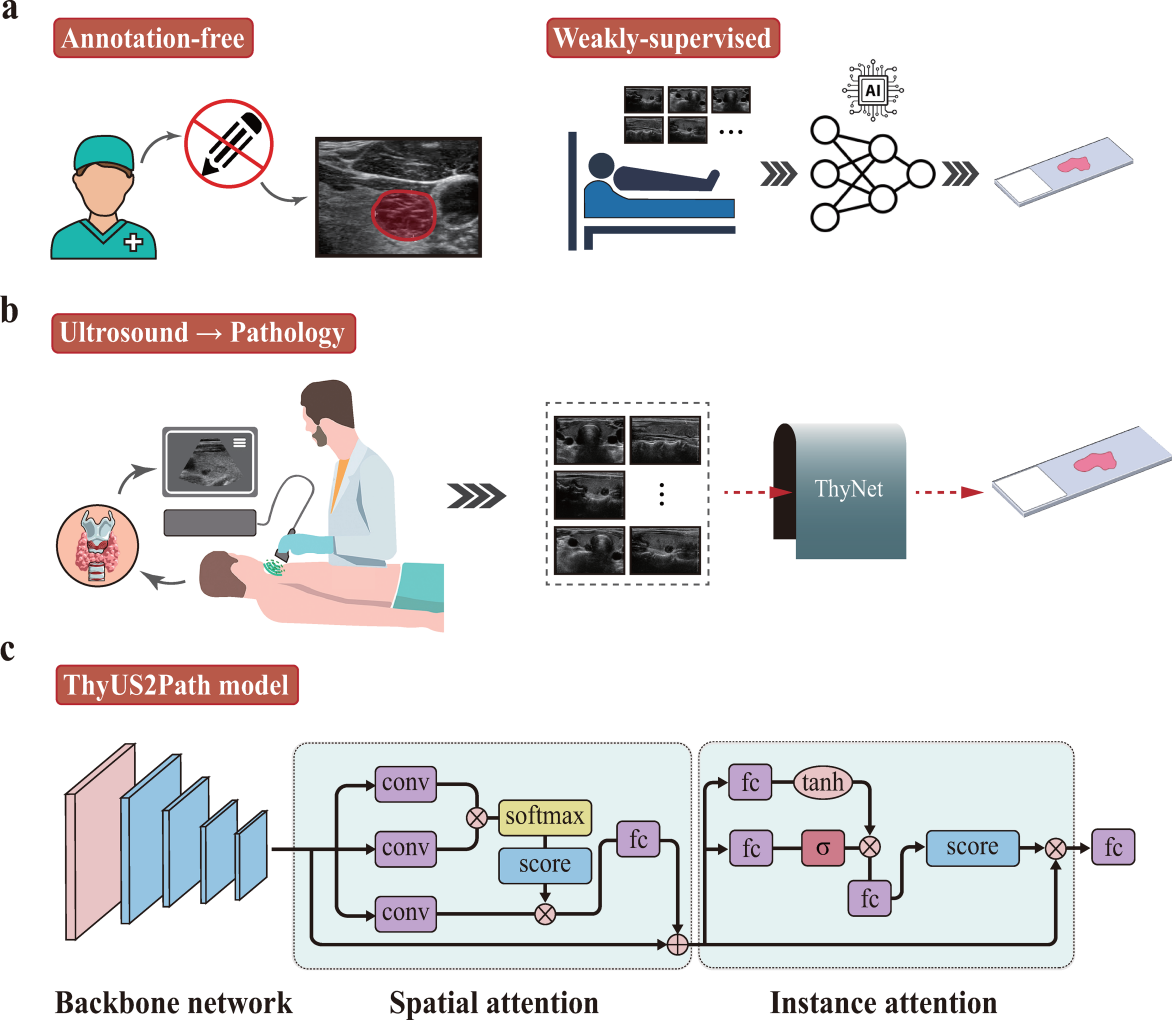
Overview of the proposed deep learning framework in this study. **(a, b)** Strategy for training. Multiple thyroid images of each patient are put into ThyUS2Path to obtain the histological result. **(c)** The proposed framework consists of backbone network, dual-attention module and fully-connected classifier.

Our model consists of three interconnected modules (**Figure 1**):

1) Backbone network: we modified the state-of-the-art ResNet-34 as our backbone network to extract relevant features from each thyroid image. For ResNet-34, we removed the last classification layer, that is, the fully connected layer with 1000 neurons. Thyroid images of each patient were feed into the backbone network to automatically extract tumor-related nodule patterns.

2) Dual attention feature aggregation module: the thyroid nodule features extracted by the backbone network were forwarded into a dual-attention module (**Figure 1**). The detailed structure of the module is as follows:

The dual-attention module consists of two sub-modules, that is, spatial attention and instance attention, respectively. We selected the output of last convolutional layer of the backbone network as input of the dual-attention module. Firstly, the input feature of several images of each patient was filtered along spatial dimension by the spatial attention to capture the subtle relationship between adjacent regions of each image. Importance scores of each region were generated to measure its contributions to the final target. Then, the filtered features were forwarded into the instance attention module. This process weighted different images of each case using attention scores and aggregated them into patient-level thyroid nodule feature representation.

3) Fully connected layer classifier: the last step consists of a fully connected layer of 2 neurons. This step transforms the patient-level features generated by the previous dual-attention module into a patient-level histological diagnosis prediction.

To improve methodological clarity, we summarize the end-to-end workflow as follows: (i) construct patient-level bags from all available ultrasound images, (ii) extract image-level deep features using the ResNet-34 backbone, (iii) calibrate intranodular spatial saliency and inter-image diagnostic importance via dual attention, and (iv) output patient-level benign/malignant prediction.

### Implementation details and training workflow

To provide a clearer and more rigorous description of the proposed framework, the mathematical formulation and workflow are summarized as follows.

For the i-th patient, let a bag be denoted as ${\text{B}}_{\text{i}}={\left\{{\text{x}}_{\text{ij}}\right\}}_{\text{j}=1}^{{\text{n}}_{\text{i}}}$, where ${\text{x}}_{\text{ij}}$ is the j-th ultrasound image and ${\text{n}}_{\text{i}}$ is the number of images for that patient. The bag-level label is ${\text{y}}_{\text{i}}\in \{0,1\}$, where 1 denotes malignant and 0 denotes benign.

Step 1 — Backbone Feature Extraction:

Each instance ${\text{x}}_{\text{ij}}$ is encoded by the ResNet-34 backbone ${\text{f}}_{\text{θ}}(\cdot )$ (with the final FC layer removed) into a spatial feature map:


$${\text{h}}_{\text{ij}}={\text{f}}_{\text{θ}}({\text{x}}_{\text{ij}})\in {\text{R}}^{\text{C}\times \text{H}\times \text{W}}$$

Step 2 — Spatial Attention Module (IRAM):

The spatial attention module refines ${\text{h}}_{\text{ij}}$ by capturing inter-regional dependencies. Three parallel convolutional projections ${\text{ψ}}_{1},{\text{ψ}}_{2},{\text{ψ}}_{3}:{\text{R}}^{\text{C}\times \text{H}\times \text{W}}\to {\text{R}}^{{\text{C}}^{\prime }\times \text{H}\times \text{W}}$ (with ${\text{C}}^{\prime }=128$) are applied. A downsampled key/value map ${\tilde{\text{h}}}_{\text{ij}}$ is obtained via bilinear interpolation at scale 0.5. The attention map is:


$${\text{Ψ}}_{12}=\text{softmax}(\frac{{\text{ψ}}_{1}{\left({\text{h}}_{\text{ij}}\right)}^{⊤}\cdot {\text{ψ}}_{2}({\tilde{\text{h}}}_{\text{ij}})}{\text{τ}})$$

where ${\tilde{\text{h}}}_{\text{ij}}$ is the downsampled feature and τ is a scaling factor. The refined feature is obtained via a residual connection:


$${\tilde{\text{h}}}_{\text{ij}}={\text{ψ}}_{4}({\text{Ψ}}_{12}\cdot {\text{ψ}}_{3}({\tilde{\text{h}}}_{\text{ij}}))+{\text{h}}_{\text{ij}}$$

This residual formulation ensures that the spatial attention acts as a feature enhancement rather than a replacement, preserving low-level information.

Step 3 — Instance Attention Module (ISAM):

The spatially refined features ${\tilde{\text{h}}}_{\text{ij}}$ are globally average-pooled to obtain instance-level vectors ${\text{v}}_{\text{ij}}\in {\text{R}}^{\text{C}}$. A gated attention mechanism then computes instance importance weights:


$${\text{a}}_{\text{ij}}={\text{w}}_{\text{c}}^{⊤}(\text{tanh}({\text{W}}_{\text{a}}{\text{v}}_{\text{ij}})⊙\text{σ}({\text{W}}_{\text{b}}{\text{v}}_{\text{ij}}))$$

$${\text{α}}_{\text{ij}}=\frac{\text{exp}({\text{a}}_{\text{ij}})}{\sum_{\text{k}=1}^{{\text{n}}_{\text{i}}}\text{exp}({\text{a}}_{\text{ik}})}$$

where ${\text{W}}_{\text{a}},{\text{W}}_{\text{b}}\in {\text{R}}^{\text{D}\times \text{C}}$ ( D=256) and ${\text{w}}_{\text{c}}\in {\text{R}}^{\text{D}\times 1}$ are learnable parameters, and ⊙ denotes element-wise multiplication. The bag-level representation is the attention-weighted sum:


$${\text{z}}_{\text{i}}=\overset{{\text{n}}_{\text{i}}}{\underset{\text{j}=1}{\sum }}{\text{α}}_{\text{ij}}\;{\text{v}}_{\text{ij}}\in {\text{R}}^{\text{C}}$$

Step 4 — Classification:

The bag-level representation ${\text{z}}_{\text{i}}$ is passed through a fully connected layer followed by a softmax function to produce the predicted class probabilities:


$${\text{p}}_{\text{i}}=\text{softmax}({\text{W}}^{⊤}{\text{z}}_{\text{i}}+\text{b})\in {\text{R}}^{2}$$

where $\text{W}\in {\text{R}}^{\text{C}\times 2}$ and $\text{b}\in {\text{R}}^{2}$ are learnable parameters, and ${\text{p}}_{\text{i},1}$ denotes the predicted malignancy probability.

Step 5 — Loss Function:

Model parameters are optimized by minimizing the standard cross-entropy loss over all N training patients:


$$\text{L}=-\frac{1}{\text{N}}\overset{\text{N}}{\underset{\text{i}=1}{\sum }}{\text{logp}}_{\text{i,}\;{\text{y}}_{\text{i}}}$$

where ${\text{p}}_{\text{i},{\text{y}}_{\text{i}}}$ is the predicted probability assigned to the ground-truth class ${\text{y}}_{\text{i}}$.

### Implementation details

To facilitate reproducibility and practical implementation, the key training settings are detailed below.

Backbone: ResNet-34 pretrained on ImageNet; final FC layer removed; output feature map $\in {\text{R}}^{512\times \text{H}\times \text{W}}$IRAM: C=512, ${\text{C}}^{\prime }=128$, τ=128, dropout p=0.25ISAM: L=512, D=256, n_classes=2, dropout p=0.25Optimizer: SGD (momentum = 0.9, weight decay = $5\times 10^{-4}$); learning rate = $1\times 10^{-3}$ with cosine warm-up over the first epoch (warm-up factor = 1/1000) and MultiStepLR decay (milestones: epochs 50, 75; γ=0.1).

Loss function: Cross-entropy loss (2-class)

Batch size: 10 patients per batch (padded to equal instance count via zero-padding)

Training epochs: 100; best model selected by validation AUROC

Data augmentation (training only): random horizontal flip ( p=0.5), random rotation (0°/90°/180°/270°)

Input normalization: resize to 256×256, normalize with ImageNet mean/std ( μ=[0.485,0.456,0.406], σ=[0.229,0.224,0.225]).

Hardware: Two NVIDIA Quadro GV100–32 GB GPUs; PyTorch v1.6.

### Comparison to baseline methods

We compared ThyUS2Path with two commonly used state-of-the-art MIL-based methods: Meanpool and Maxpool. In Meanpool, the features of n images in a bag were averaged to obtain an aggregated patient-level representation, while Maxpool used max operation to aggregate image-level features. Both of them were trained on the same model architecture as ThyUSPath, except for replacing the dual-attention module by mean and max operation, respectively.

### Statistical analysis

Patient-level AUROC (area under the ROC curve) and AUPRC (area under the PR curve) were used to measure model performance. All experiments were performed using a workstation equipped with two NVIDIA Quadro GV100 32GB GPU cards. The model was implemented using the PyTorch (version 1.6) framework. The statistical analysis was conducted in R (version 4.0.3) by using pROC (version 1.17.0.1) for receiver operating characteristic (ROC) statistics. In addition to AUROC and AUPRC, we evaluated sensitivity, specificity, positive predictive value (PPV), and negative predictive value (NPV) at a predefined operating point. These clinically relevant metrics were calculated on a per-fold basis. All the plots were made using the matplotlib package in Python.

## Results

### Characteristics of images and patients

**Figure 2:**
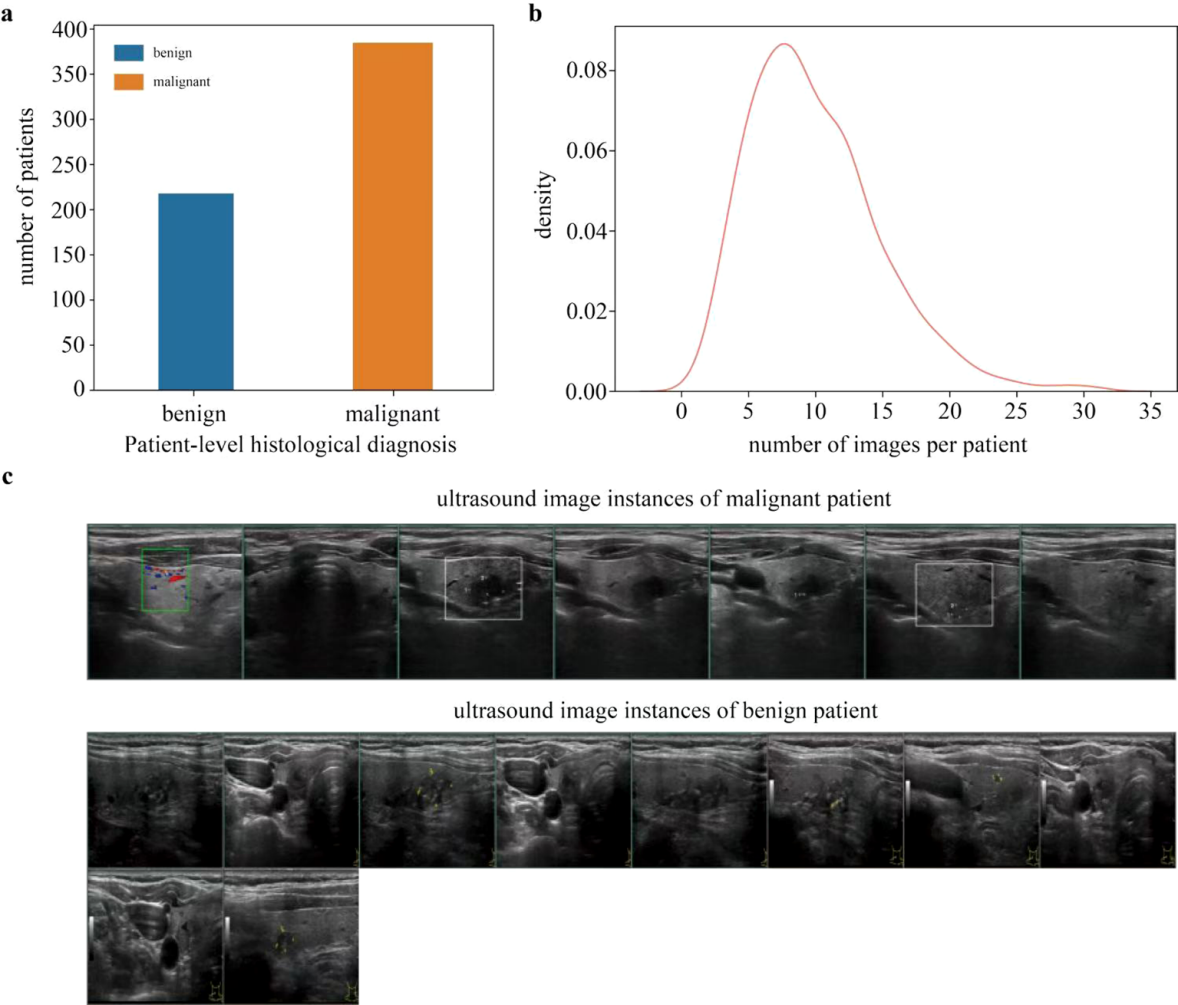
Characteristics of the training dataset and representative ultrasound image instances. **(a)** Distribution of patient-level histopathological labels (benign vs. malignant) in the training cohort. **(b)** Distribution of the number of ultrasound images per patient in the training cohort. **(c)** Representative thyroid ultrasound image instances from different patients, illustrating inter-case and intra-case imaging heterogeneity.

We used 6014 thyroid images from 603 patients to build the deep learning framework. The dataset characteristics were summarized in **Figure 2**. As shown in **Figure 2A**, the training dataset was consisted of 603 patients, including 218 benign ones and 385 malignant ones with histological reports. Since each patient may have multiple thyroid images, we made a statistical description of all these patients in **Figure 2B**. We also displayed some thyroid nodule images from malignant patient and benign patient, respectively.

### Classification performance of ThyUS2Path

**Figure 3:**
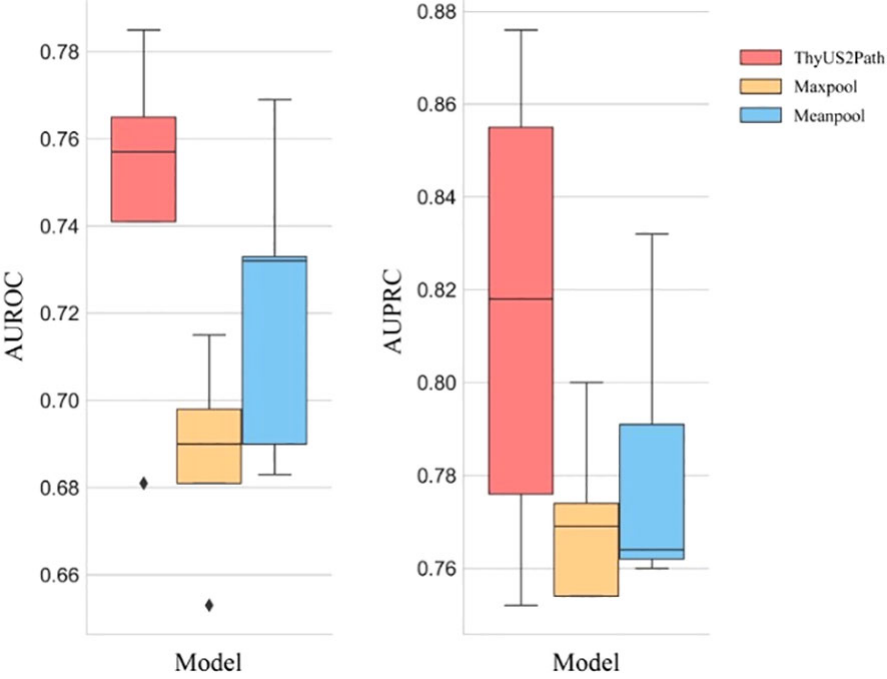
Comparison of the performance between ThyUS2Path and state-of-the-art MIL-based methods on internal test set.

After the training of ThyUS2Path, we validated the five-fold cross validation model performance on internal testing dataset of 548 thyroid images. The ThyUS2Path yielded an average test AUROC of 0.745 ± 0.035, and an average test AUPRC of 0.830 ± 0.047 (**Figure 3**), demonstrating the efficacy of the proposed approach for the histological determination of malignant and benign thyroid nodules directly from ultrasonography. To further evaluate model performance, we compared ThyUS2Path with two commonly used state-of-the-art MIL-based methods, that is, Maxpool and Meanpool, respectively. For a fair comparison, we used the same backbone feature extractor in all methods. Comparison results on internal test set were displayed in **Figure 3**. At the predefined operating point, fold-wise diagnostic metrics were as follows:sensitivity (0.7213, 0.5735, 0.4667, 0.8056, 0.8310), specificity (0.7660, 0.8293, 0.9412, 0.5833, 0.6757), PPV (0.8000, 0.8478, 0.9459, 0.7945, 0.8310), and NPV (0.6792, 0.5397, 0.4444, 0.6000, 0.7778) across folds 1-5, respectively (**Supplementary Table 1**). Overall, ThyUS2Path outperformed all the other approaches significantly.

### Generalizability to external test cohort

Due to the variation caused by different operators and different protocols for thyroid ultrasonography, images from different cohorts could vary in appearance. It is crucial to ensure the generalizability of computation ultrasonography algorithms to real-world data in clinical practice. We collected a total of 1978 thyroid nodule images of another cohort as an external test dataset. As demonstrated in **Table 1**, the model achieved encouraging performance in this temporally independent cohort (AUROCs of 0.70-0.80 and AUPRCs of 0.78-0.83), supporting preliminary external robustness within the same hospital system. Using the same ResNet-34 backbone and training protocol, ThyUS2Path (dual-attention aggregation) showed improved and more stable external performance than non-attention MIL baselines (Meanpool/Maxpool), supporting the benefit of attention-based feature aggregation at the module level.

**Table 1:** Model performance of five-fold cross validation on the external test set.

Model	Fold	AUROC^*^	AUPRC^*^
ThyUS2Path	Fold1	0.738	0.800
	Fold2	0.708	0.777
	Fold3	0.718	0.798
	Fold4	0.724	0.817
	Fold5	0.790	0.834
Maxpool	Fold1	0.805	0.849
	Fold2	0.652	0.776
	Fold3	0.722	0.810
	Fold4	0.757	0.846
	Fold5	0.691	0.790
Meanpool	Fold1	0.647	0.742
	Fold2	0.668	0.759
	Fold3	0.693	0.750
	Fold4	0.653	0.749
	Fold5	0.737	0.797

^*^ AUROC, area under the ROC curve; AUPRC, area under the PR curve.

### Visualization results

**Figure 4:**
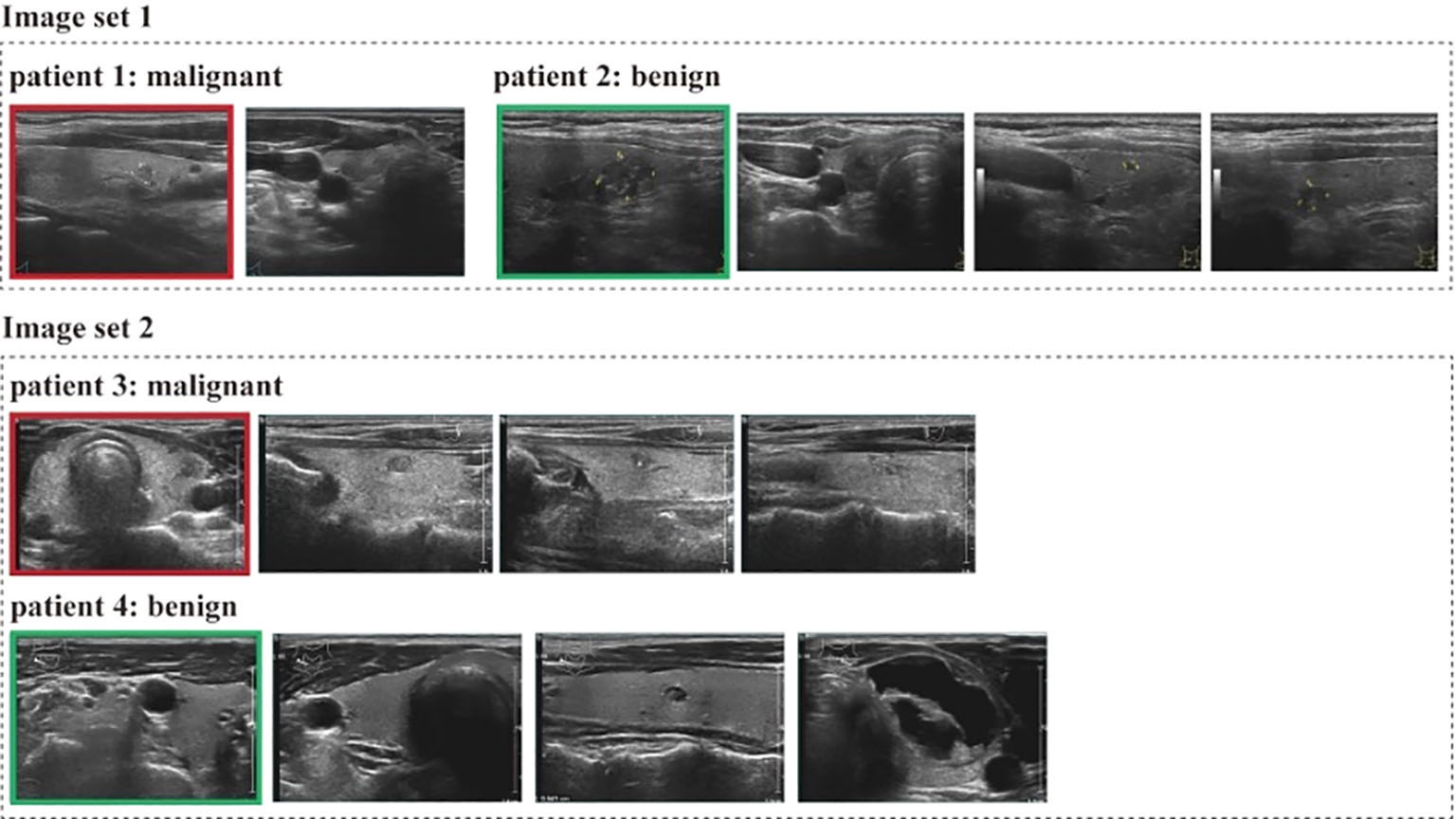
Top images (red box) and tail images (green box) weighted by the attention scores generated by the dual-attention module.

Interpretability is important for human to understand the internal predictive mechanism of deep learning models. We sorted the attention scores generated by the instance attention module in ThyUS2Path and selected the top images for clinicians to make a explainable diagnosis. As shown in **Figure 4**, four patients were randomly selected from the test set and were divided into two image sets, that is, Image set 1 and Image set 2. Then images in each image set were put into ThyUS2Path to get the corresponding attention scores. We found that the top images were exactly in malignant patients for both images set 1 and image set 2. It indicated that the attention module in ThyUS2Path could identify the most discriminative nodule patterns. To improve interpretability assessment, we additionally analyzed failure cases (false positives and false negatives). False negatives were often associated with nodules showing less typical malignant sonographic appearances, while false positives frequently occurred in artifact-prone images with suspicious-like echo patterns. These findings indicate that the model’s failure modes are broadly aligned with known diagnostic challenges in thyroid ultrasonography. A quantitative concordance analysis between attention maps and radiologist-annotated image-level features was not feasible in this retrospective cohort due to lack of standardized expert pixel/region annotations.

## Discussion

US is a widely used technique for thyroid nodule diagnosis, and actual clinical studies are often confronted with many US images from the same nodule or multiple nodules of a patient, corresponding to the pathological diagnosis of a FNAB of a particular nodule. In this study, we created a novel dual-attention guided deep learning framework, ThyUS2Path, to provide an automated recognition scheme for ultrasound diagnosis of thyroid nodules. The proposed method avoided detailed image-level annotations and could predict pathological results directly from US images. The findings suggest that ThyUS2Path can enable linking US phenotypes to histological reports in a non-invasive manner to enhance thyroid cancer diagnosis and avoid unnecessary FNAB, while avoiding large-scale image evaluation.

We believe that directly improving the accuracy of US for thyroid cancer diagnosis can minimize costs and detect those occult lesions, as it can suggestively guide the need for FNAB and reduce the impact of operator variation. The variable appearance and blurred borders of thyroid nodules make it difficult for radiologists to make an accurate identification and consistent interpretation of malignant nodules (30). Deep learning has the advantage of overcoming the problem of heterogeneity and can learn feature representations automatically, and in some clinical outcome studies, deep learning algorithms outperformed clinicians (24, 25). Recent advances in endocrine oncology and clinical-grade medical AI further support the development of robust, clinically translatable thyroid diagnostic models (31, 32). In particular, convolutional neural networks have been widely used to learn imaging features for optimal pattern recognition performance. Convolutional neural networks have also achieved good diagnostic performance in studies using ultrasound images to diagnose the malignancy of thyroid nodules. For example, Ma and colleagues proposed a method for thyroid nodule diagnosis that incorporates two pre-trained convolutional neural networks with different convolutional and fully connected layers, and analyzed 8148 manually labeled thyroid nodules to obtain a diagnostic accuracy of 83.0% (95% CI 82.3-83.7) for thyroid nodules (33). Xia and colleagues explored the performance of an extreme learning machine trained using radiologist-annotated features, and the study achieved 87.7% accuracy in distinguishing malignant from benign nodules using 203 ultrasound images from 187 thyroid cancer patients (34).

A single ultrasound image can provide partial anatomical information of the thyroid gland, and multiple ultrasound images of the same thyroid nodule from different angles and different nodules in the same patient are often acquired during ultrasound examinations. In actual clinical practice, there is often a lack of image-level annotations, which hindered the effective training of deep learning models for thyroid nodule classification. To solve the above problems, a classification model can be constructed using multi-instance learning strategy to potentially achieve robust classification performance using ultrasound images from different views of the same patient (35, 36).

Different from the previous multi-instance learning-based methods, the proposed ThyUS2Path model made several improvements. For instance, we designed a dual-attention module, consisting of spatial attention and instance attention, to learn more effective and accurate nodule feature representations. Specifically, the spatial attention module was developed to calibrate the nodule image features along the spatial dimension. This operation could comprehensively integrate regional features in US images and focus on more important image features for diagnosis. In addition, the instance attention module was used to further calibrate the extracted features along the channel dimension. It was able to integrate multi-scale nodule features.

Our study has several limitations. First, this study relied primarily on data sets from the same hospital sources. Although an independent temporal cohort was used for external validation, both cohorts were derived from the same hospital system. Therefore, the current results may not fully capture inter-center heterogeneity, including operator-dependent scanning behavior, ultrasound vendor differences, and acquisition protocol variability. Prospective multi-center validation across geographically distinct institutions and different ultrasound platforms is required before broad clinical deployment. Second, all study samples were derived from patients who underwent pathological examination in a hospital setting rather than screening scenario. Different prevalence rates may significantly affect the positive and negative predictive values between populations, which may reduce the potential generalizability of the results. Third, a direct comparison with radiologist performance and TIRADS-based classification was not conducted in this retrospective analysis, because standardized paired reader-level interpretations and harmonized TIRADS records were not consistently available for all included cases. Future prospective studies should incorporate head-to-head reader-study designs to evaluate clinical utility relative to routine diagnostic standards. Fourth, our patient-level labeling strategy may introduce label noise in cases with mixed benign and malignant nodules. Nevertheless, this strategy reflects a clinically pragmatic decision rule, as the presence of any malignant nodule usually determines downstream intervention.

## Conclusion

Improving ultrasonography to correctly categorize thyroid nodules is important for clinical decision making, as it can guide subsequent treatment. Our algorithm has the potential to directly enable the interpretation of thyroid cancer at the individual level without the need for ultrasonography labeling and feature extraction. If further validated in prospective multi-center cohorts, the algorithm may help reduce unnecessary invasive biopsies and support clinical decision-making in thyroid nodule management.

## Data Availability

The raw data supporting the conclusions of this article will be made available by the authors, without undue reservation.

## Supplementary material

Supplementary Table 1 Fold-wise clinically relevant diagnostic metrics of ThyUS2Path at the predefined operating point. PPV = positive predictive value, NPV = negative predictive value
