# Supplementary material for: Pathological diagnosis of thyroid nodules directly from ultrasonography by a weakly supervised deep learning framework
Source: Front Endocrinol (Lausanne). 2026 Jun 1;17:1834977. doi: 10.3389/fendo.2026.1834977 (PMC13231192; doi:10.3389/fendo.2026.1834977)
Supplement: Supplementary Table 1 — Fold-wise clinically relevant diagnostic metrics of ThyUS2Path at the predefined operating point. PPV = positive predictive value, NPV = negative predictive value [file Table1.docx]

**Supplementary Table 1**. Fold-wise clinically relevant diagnostic metrics of ThyUS2Path at the predefined operating point

|  | sensitivity | specificity | PPV | NPV |
| --- | --- | --- | --- | --- |
| Fold 1 | 0.7213 | 0.7660 | 0.8000 | 0.6792 |
| Fold 2 | 0.5735 | 0.8293 | 0.8478 | 0.5397 |
| Fold 3 | 0.4667 | 0.9412 | 0.9459 | 0.4444 |
| Fold 4 | 0.8056 | 0.5833 | 0.7945 | 0.6000 |
| Fold 5 | 0.8310 | 0.6757 | 0.8310 | 0.7778 |

PPV = positive predictive value, NPV = negative predictive value
